# Utilization of the multi-theory model (MTM) of health behavior change to explain health behaviors: A systematic review

**DOI:** 10.34172/hpp.42887

**Published:** 2024-07-29

**Authors:** Sidath Kapukotuwa, Tara Nerida, Kavita Batra, Manoj Sharma

**Affiliations:** ^1^Department of Social and Behavioral Health, School of Public Health, University of Nevada, Las Vegas, NV 89119, USA; ^2^Office of Research, Kirk Kerkorian School of Medicine at UNLV, University of Nevada, Las Vegas, NV 89102, USA; ^3^Department of Medical Education, Kirk Kerkorian School of Medicine at UNLV, University of Nevada, Las Vegas, NV 89102, USA; ^4^Department of Internal Medicine, Kirk Kerkorian School of Medicine at UNLV, University of Nevada, Las Vegas, NV 89102, USA

**Keywords:** Behavior, Phycological theory, Social theory, Systematic review

## Abstract

**Background:**

The utilization of a theoretical framework is vital in health promotion research, particularly when endeavoring to modify health behaviors. This systematic review aimed at evaluating and synthesizing evidence through studies conducted using the fourth-generation multi-theory model (MTM) of health behavior change for its effectiveness.

**Methods:**

A comprehensive article search was performed across MEDLINE, CINAHL, and Academic Search Premier. The search focused on studies utilizing MTM from 2016 to December 2023, following the PRISMA guidelines for systemic reviews.

**Results:**

An initial pool of 7583 articles was narrowed down through screening of titles, abstracts, and full texts. A total of 69 articles met the inclusion criteria. These studies, encompassing a global range of diverse target groups and health behaviors, were categorized as qualitative, cross-sectional, or experimental. The six qualitative studies revealed MTM themes for diverse health behaviors. The fifty-six cross-sectional studies showed MTM constructs effectively predicting behavior change, albeit with varying statistical significance. The seven experiments demonstrated MTM’s role in initiating and sustaining change. For the initiation model, operationalized by 49 studies, the mean adjusted R^2^ was 38.4% (SD=16.4%). For the sustenance model, operationalized by 45 studies, the mean adjusted R^2^ was 38.9% (SD=15.5%).

**Conclusion:**

This systematic review corroborates the MTM as a potent framework for understanding, predicting, and facilitating health behavior changes. Its universal applicability and effectiveness underscore the model’s potential as a foundational tool in designing future health promotion strategies and interventions aimed at positive and enduring behavior modifications.

## Introduction

 An individual’s health and health-related actions reflect the various physical and social factors they encounter over time.^1^ These deliberate or unintentional behaviors can either positively or negatively impact an individual’s overall health. For instance, only a small fraction of adults, just one in ten, adhere to the federal recommendations for daily fruit and vegetable consumption.^2^ Additionally, every year, tobacco consumption leads to more than 8 million deaths globally, including approximately 1.3 million non-smokers who die due to second-hand smoke exposure.^3^ While many health behaviors are preventable, advancing our understanding through research, data collection, and interventions is crucial.^1^

 Utilizing a theoretical framework is essential to provide structure and guidance for research.^4^ In the realm of public health, the utilization of a theoretical framework is pivotal for understanding and influencing health behavior. Previous studies have attempted to identify key factors influencing behavior, but predicting individual behavior in specific situations remains challenging.^5^ At the individual level, behavior change may be driven by subtle automatic responses, but at the population level, these changes contribute to patterns of health disparities.^5^ Research investigating the interplay between individual and societal patterns requires robust fourth-generation theoretical models.

 Within the realm of theoretical models, the multi-theory model (MTM) of health behavior change, introduced in 2015, offers a comprehensive framework exclusively focused on predicting and facilitating health behavior change.^6^ MTM is distinguished by its strong predictive power, simplicity, and empirically tested constructs.^6^ Furthermore, MTM is versatile, applicable across diverse cultural and socioeconomic contexts, and addresses both short-term and long-term behavior changes.^6^ Due to its unique capacity to explain behavioral intentions and persistence, MTM is considered an ideal framework for predicting and promoting health behavior change.^6^

 MTM comprises two components that facilitate health behavior change: initiation and sustenance.^6^ Initiation involves three primary constructs: participatory dialogue, behavioral confidence, and changes in the physical environment.^6^ Participatory dialogue is derived from perceived advantages and perceived disadvantages of health behavior change, and the dialogues facilitated by health educators to promote change.^6^ Behavioral confidence reflects an individual’s belief in their ability to initiate and achieve the desired behavior change, influenced by internal and external factors.^6^Changes in the physical environment encompass the tangible resources that enable individuals to initiate behavior change.^6^

 The second component of MTM, the sustenance of health behavior change, includes three key constructs: emotional transformation, practice for change, and changes in the social environment.^6^Emotional transformation involves individuals altering their emotions towards a health behavior change.^6^Practice for change entails continuous reflection on the behavior change, ongoing evaluation, and adjustment of strategies, overcoming barriers, and maintaining focus on sustaining the change.^6^Changes in the social environment encompass natural or artificial sources of social support in the environment.^6^

 In recent years, the MTM has emerged as a robust framework for understanding and promoting health behavior change across a diverse range of contexts. Notably, MTM’s applicability in predicting initiation and sustenance of health behaviors has been demonstrated across various health domains. For instance, Sharma et al applied MTM in assessing COVID-19 vaccine acceptance among college students,^7^ revealing significant predictors of vaccination behavior. Similarly, Brown et al^8^ leveraged MTM to examine the efficacy of interventions aimed at increasing fruit and vegetable consumption among African American women,^8^ showcasing the model’s effectiveness in dietary behavior change. Furthermore, studies like Bashirian et al have utilized MTM in the context of smoking cessation among adolescents,^9^ underscoring the model’s relevance in addressing addictive behaviors. These examples, among others, highlight MTM’s versatility and potent predictive capability in fostering positive health behavior changes.

 To date, there has been no comprehensive synthesis of the effectiveness and predictability of MTM in qualitative, descriptive, and experimental studies. One study by Godfrey and Colleagues in 2010 provided collective evidence on the utilization of theoretical models and frameworks, however, evidence related to the utility of the Fourth-generation models is lacking until today.^10^ Therefore, the objective of this systematic review is to evaluate and consolidate evidence from numerous studies grounded in MTM and assess the effectiveness of this theoretical framework in predicting and promoting successful behavior change, both in the short-term and long-term. Additionally, this review aims to unveil the explanatory power of the MTM framework in studying a diverse range of behaviors in different settings.

## Methods

###  Protocol

 This systematic review follows the Preferred Reporting Items for Systematic Reviews and Meta-Analyses (PRISMA) Checklist as delineated by Page et al^11^ in 2021. We have diligently integrated most of the 27 essentials reporting elements advocated by PRISMA into our methodology to systematically identify crucial concepts and pinpoint areas of knowledge deficiency.^11^ Detailed information regarding the checklist and the corresponding sections evaluated can be found in Table S1 (Supplementary file 1).

###  Eligibility criteria

 The inclusion criteria for this review encompass studies published between 2016 and December 2023, focusing on original peer-reviewed research that exclusively utilized the MTM for health behavior change for predicting or fostering behavior change. The MTM model was first introduced in 2015; therefore, it was reasonable to select the following year (2016) as the lower value of the range of the data collection window to capture evidence related to its utilization. Qualitative, descriptive-cross-sectional, and experimental studies published in the English language and conducted in any part of the world were considered for inclusion, while studies grounded in alternative theoretical frameworks, gray literature, non-peer-reviewed publications, reviews, just abstracts, editorials, commentaries, letters, opinion pieces, case reports, dissertations, presentations, and biochemical or animal studies were excluded. Additionally, articles that were not accessible were excluded from this study. For experimental studies, we used the following research question to guide our literature search, “Among diverse populations across various settings, how effective is the MTM of health behavior change compared to other theoretical models or baseline measurements in predicting and promoting the initiation and sustenance of health behaviors?” These criteria were employed to ensure the selection of pertinent and rigorously conducted research for the systematic review.

###  Information sources and search

 The search strategy employed for this review incorporated various key terms, including ‘MTM’, ‘multi-theory model’, ‘multitheory model’, ‘multi theory model’, ‘Initiation’, ‘Sustenance’, and ‘Health behaviors’, encompassing both ‘Obesogenic behaviors’ and ‘Human behavior’. To ensure comprehensive coverage, we used a combination of Boolean operators (AND, OR), truncation, and MeSH terms. This inclusive approach, without specifying particular health behaviors, aimed to encompass a wide range of studies that have employed MTM. The search was conducted across multiple sources, including MEDLINE (PubMed), CINAHL, and Academic Search Premier, to capture a diverse array of relevant studies.

###  Selection of studies

**Figure 1:**
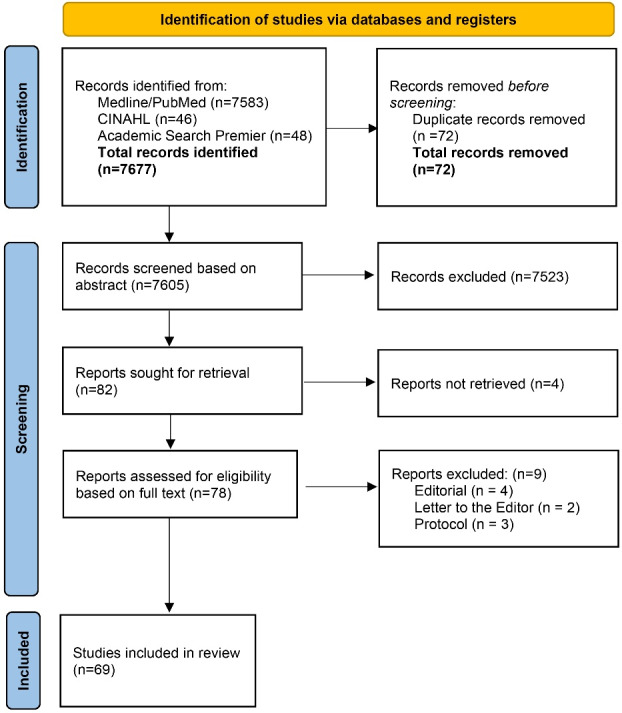
PRISMA 2020 flow diagram of MTM studies identified

 Articles were collected and subjected to a screening process following the PRISMA flow diagram (Figure 1). This screening involved a sequential evaluation of titles, abstracts, and full texts to determine their eligibility for potential inclusion or exclusion. Ultimately, articles that centered on the utilization of MTM for the examination of various aspects of human health behavior were retained for further review. Reviewers SK and TN participated independently in this comprehensive review process. The analysis revealed a remarkably high level of agreement between the two researchers, characterized by an inter-rater agreement of 98%. If there was a disagreement, the decision was made by MS.

###  Data extraction

 The chosen articles for inclusion underwent a subsequent stage of data extraction. Two reviewers (SK and TN) independently participated in the data extraction. In the process of full-text screening, nine studies were removed from consideration for this review because they were identified as editorials,^12-15^ letters to the editor,^16,17^ or study protocols.^18-20^ These excluded studies are detailed in Table S2. Information extracted from these articles was further categorized into three distinct groups based on the research study design employed: 1) qualitative studies; 2) descriptive, cross-sectional studies; and 3) experimental studies. This systematic review encompasses six qualitative studies, fifty-six cross-sectional studies, and seven experimental studies, culminating in a total of 69 studies. The data was then summarized to encompass several key data points, including the author’s name and publication year, study location, type of behavior studied, the sample characteristics, the specific study design employed, and the main findings. Key predictors (MTM constructs) of qualitative studies, variances of initiation and sustenance models in cross-sectional studies, and significant findings in experimental studies are presented in the main findings.

###  Study risk of bias assessment

 For the risk of bias assessment in experimental studies, distinct methodologies were employed based on the study design. Randomized controlled trials (RCTs) were evaluated using the Cochrane risk-of-bias tool for randomized trials (RoB 2).^21^ In contrast, quasi-experimental and non-randomized control trial studies were assessed using the Cochrane risk-of-bias tool for non-randomized studies of interventions (ROBINS-I).^22^ The outcomes of these assessments are detailed in Table 1. SK completed this comprehensive assessment process.

## Results

###  Qualitative studies

 Six articles were identified as qualitative studies employing MTM. These studies employed various methods for data collection, such as individualized interviews, focus group discussions, and journal entries. Among them, two studies focused on smoking cessation behavior,^28,29^ two studies addressed vaccination acceptance behavior,^30,31^ one study examined drug use relapse behavior,^32^ and one study explored yoga behaviors.^33^ These investigations revealed that MTM constructs effectively predicted the initiation and continuity of health behavior change, thereby offering valuable insights for the development of effective behavioral interventions. Detailed information on the qualitative studies can be referred to in Table 2.

 The qualitative studies detailed in Table 2 underscore the MTM’s comprehensive applicability across varied health behaviors and contexts, highlighting pivotal aspects of health behavior change. Central to these findings is the role of behavioral confidence and social support. Studies such as Bashirian et al^28^ on waterpipe smoking reduction among Iranian high school students reveal that an individual’s belief in their ability to change,^28^ coupled with robust social support, is crucial in both initiating and maintaining health behavior changes. This aligns with the MTM’s emphasis on participatory dialogue and the social environment’s modification as key constructs for behavior change.

 Additionally, addressing misconceptions and enhancing knowledge emerge as vital components in effecting health behavior change. For instance, Barolia et al^29^ on cigarette smoking cessation and Agyei-Baffour et al^30^ on HPV vaccination practices among Ghanaian healthcare providers illustrate the necessity of tailored educational interventions. These interventions are essential to overcome barriers related to misinformation and gaps in knowledge, further emphasizing the need for contextually relevant health promotion strategies.

 The findings also highlight the significance of emotional transformation and changes in the physical and social environments in supporting health behavior change. Mousali et al^32^ identified these factors as main predictors of relapse to drug use among Iranian male addicts, indicating that interventions need to extend beyond individual factors to include emotional well-being and environmental modifications. This suggests a paradigm shift towards more holistic approaches in health behavior change, considering the interplay of emotional, environmental, and social dynamics.

 Cultural and contextual relevance also plays a pivotal role in shaping health behaviors, as evidenced by studies such as Su et al^31^ on COVID-19 vaccination willingness among Chinese young adults. This study, among others, highlights how societal expectations, policy requirements, and perceptions of social responsibility influence individuals’ health-related decisions and actions. It underscores the importance of understanding and integrating cultural and contextual nuances into health behavior change interventions.

 In essence, the application of the MTM across diverse settings and behaviors demonstrates its versatility and effectiveness as a framework for health behavior change. The qualitative studies provide valuable insights into the multifaceted nature of health behavior change, advocating for interventions that are not only multi-theoretical but also culturally and contextually tailored. This comprehensive approach, which addresses behavioral, emotional, environmental, and social factors, underscores the MTM’s potential in guiding effective health promotion and behavior change strategies.

###  Descriptive, cross-sectional studies

 A total of 56 articles were identified as cross-sectional studies employing MTM. Nine studies focused on vaccination related behaviors.^7,34-41^ Eight studies focused on healthy eating related behaviors.^42-49^ Seven studies focused on physical activity behaviors.^50-56^ Four studies focused on smoking/vaping related behaviors.^57-60^Three studies each focused on following the behaviors of: stress management behaviors,^61-63^ use of new technology related behaviors,^64-66^ and oral hygiene related behaviors.^67-69^ Two studies each focused on the following behaviors of: adequate sleep behaviors,^70,71^ sugar-sweetened beverage consumption related behaviors,^72,73^ mammography screening related behaviors,^74,75^ and cancer screening behaviors.^76,77^ One study each focused on the following behaviors of: responsible drinking behavior,^78^ substance use behavior,^79^ handwashing behavior,^80^ positive airway pressure therapy behavior,^81^ mask-wearing behavior,^82^ sunscreen usage behavior,^83^ meditation intention related behavior,^84^ yoga practice behavior,^85^ screen time behavior,^86^ indoor tanning cessation behavior,^87^ and gambling behavior.^88^ Much like the qualitative studies, these articles shared the objective of forecasting both the initiation and sustenance of behavior changes as outlined above. Comprehensive information regarding the data from these descriptive studies can be found in Table S3.

 Among the 56 articles, researchers utilized MTM-based questionnaires, administered either in-person with paper and pencil or online, with varying numbers of items tailored to reflect the specific health behavior under investigation, the characteristics of the target population, and the predictors of both initiation and sustenance of behavior change. A majority of the studies, precisely 50, aimed to evaluate how the MTM-based questionnaire predicted both the initiation and sustenance of the studied behavior change, while also identifying statistically significant constructs associated with predicting behavior change. The remaining six studies,^35-37,39,40,68^ employed an MTM-based questionnaire solely for predicting the initiation of behavior change. Although the specific constructs predicting behavior change varied across the 56 articles, the results consistently demonstrated that at least one construct either from initiation or sustenance components was statistically significant in predicting behavior change. All studies also emphasized that practical implications may involve integrating the identified statistically significant MTM constructs into intervention design and operationalizing these constructs.

 In analyzing the effectiveness of the MTM constructs across the included studies, it becomes evident that MTM offers a robust framework for understanding health behavior change. In the initiation model, which was operationalized by 49 studies, the mean adjusted R^2^ was 38.4% (SD = 16.4%), with a range from 3.9% to 73.6% (with 34 studies reporting values in the 30s). For the sustenance model, operationalized by 45 studies, the mean explanatory potential, as indicated by adjusted R^2^, was 38.9% (SD = 15.5%), with a range from 5.3% to 73.6% (again, with 34 studies reporting values in the 30s).

 The descriptive and cross-sectional studies utilizing the MTM of health behavior change, as summarized in Table S3, provide a broad overview of the model’s applicability across a spectrum of health behaviors and populations from 2016 to December 2023. These studies collectively reinforce the MTM’s utility in predicting and influencing both the initiation and sustenance of health behavior changes in diverse contexts.

 A recurring theme across the studies is the significance of the MTM constructs—such as behavioral confidence, changes in the physical and social environments, and emotional transformation—in shaping health behavior changes. For instance, a study conducted by Asare et al^34^ on HPV vaccination among adolescents in Ghana demonstrated that perceived beliefs and changes in the physical environment were critical in initiating vaccination behavior. This is consistent with the findings from Sharma et al^7^ on COVID-19 vaccine acceptance among college students in the Southern USA, where behavioral confidence and participatory dialogue emerged as significant predictors of vaccine acceptance. These studies highlight the importance of enhancing self-efficacy and leveraging environmental modifications to promote health behavior change.

 The variance explained by the MTM constructs in predicting health behaviors varied across studies, indicating the model’s flexibility and adaptability. For example, Popelsky et al^38^ applied the MTM to understand HPV vaccination attitudes among Ghanaian parents, finding changes in the physical and social environments to be substantial predictors of vaccination behavior. This variation in predictive power across different health behaviors and contexts underscores the MTM’s comprehensive applicability, highlighting its potential as a robust framework for developing and implementing targeted health behavior change interventions.

 These findings from the descriptive and cross-sectional studies affirm the MTM’s effectiveness and versatility in health behavior change research. The model’s constructs provide a solid foundation for predicting and facilitating health behavior changes, with their significance further emphasized by the need for interventions that are both multi-theoretical and tailored to specific cultural and contextual settings. These insights contribute valuable knowledge towards advancing health promotion efforts and designing more effective behavior change interventions.

###  Experimental studies

 Seven articles were identified as experimental studies employing MTM. Among these studies, four employed randomized control trial designs,^8,9,23,24^ two utilized quasi-experimental study designs,^25,26^ and one used a non-randomized, uncontrolled design.^27^ These studies targeted various behaviors, including initiating and sustaining physical activity among African American women,^23^ reducing water pipe smoking (WPS) among male adolescent students,^8^ increasing fruit and vegetable consumption among African American women,^9^ assessing the impact of specific educational interventions on the quality of life among postmenopausal women,^24^reducing sugar consumption to decrease body mass index (BMI) and abdominal obesity in women,^25^ studying portion size consumption behavior and nutritional status among college students,^26^ andtobacco cessation among users visiting a dental college.^27^ A detailed breakdown of the data from these experimental studies is displayed in Table 3.

 The experimental studies presented in Table 3 offer robust evidence supporting the MTM’s effectiveness in initiating and sustaining health behavior changes. These studies, conducted across various health domains and populations, showcase the MTM’s applicability and efficacy in designing and implementing health interventions.

 A key observation from these studies is the significant impact of MTM-based interventions on both the initiation and sustenance of targeted health behaviors. For example, an experimental study by Hayes et al^23^ successfully demonstrated the model’s utility in increasing physical activity among African American women. This study, along with others like Yoshany et al^24^ which assessed the quality-of-life improvements in Iranian postmenopausal women through exercise, highlights the MTM’s capacity to effect meaningful change in health behaviors and outcomes.

 The experimental designs varied across the studies, including randomized control trials, quasi-experimental setups, and non-randomized uncontrolled trials, each contributing unique insights into the MTM’s operationalization and impact. Notably, these studies consistently reported positive outcomes in behavior change, underscoring the MTM constructs’ pivotal role in achieving these changes. For instance, Joveini et al^25^ focused on reducing sugar consumption to decrease BMI and abdominal obesity in women, illustrating how specific MTM constructs can be targeted to address distinct health challenges.

 Furthermore, these experimental studies underscore the model’s versatility across different behavioral interventions and populations. Whether addressing dietary habits, physical activity, or other health behaviors, the MTM provides a solid theoretical foundation for designing interventions that are both effective and adaptable to the needs and contexts of specific target groups. In essence, the findings from the experimental studies in Table 3 affirm the MTM’s value as a theoretical framework for health behavior change interventions. By demonstrating significant improvements in health behaviors through MTM-based interventions, these studies reinforce the model’s relevance and potential for guiding future health promotion efforts. This body of work not only validates the MTM’s theoretical underpinnings but also highlights its practical applicability in achieving tangible health behavior changes, offering promising directions for future research and intervention design.

**Table 1:** Risk of bias assessment of experimental studies

**Randomized control trial**								
**Author & Publication year**	**D1**	**D2**	**D3**	**D4**	**D5**	**Overall**		
Brown et al, 2020^8^	!	+	+	+	+	!		
Bashirian et al, 2019^9^	!	+	+	+	+	!		
Hayes et al, 2019^23^	!	+	+	+	+	!		
Yoshany et al, 2021^24^	!	+	+	+	+	!		
**Quasi-experimental studies**								
	**Confounding**	**Selection of participants**	**Classification of interventions**	**Deviations from intended interventions**	**Missing data**	**Measurement of outcomes**	**Reported result**	**Overall**
Joveini et al, 2023^25^	Low	Low	Low	Low	Low	Moderate	Low	Moderate
Gupta et at, 2023^26^	Low	Low	Low	Low	Low	Moderate	Low	Moderate
**Non-randomized uncontrolled trial**								
Kumar et al, 2021^27^	Low	Low	Low	Low	Low	Moderate	Low	Moderate

+ Low risk; ! Some concerns; - High risk; D1: Randomization process; D2: Deviations from the intended interventions; D3: Missing outcome data; D4: Measurement of the outcome; D5: Selection of the reported result.

**Table 2:** Qualitative studies utilizing the multi-theory model (MTM) of health behavior change from 2016 to December 2023

**Author & Publication year **	**Location of study**	**Type of behavior studied **	**Target group (setting)**	**Type of study/design**	**Main findings **
Bashirian et al, 2019^28^	Hamadan, Iran.	The study focused on the reduction of waterpipe smoking among high school students.	Male high school students in Iran, who had experienced waterpipe smoking in the last month. The study included 34 participants.	Qualitative study using directed content analysis.	The study explored factors influencing waterpipe smoking reduction among high school male students in Iran. Key predictors identified include behavioral confidence, social environment change, and participatory dialogue. Students' belief in their ability, support from friends, and understanding of the benefits of waterpipe smoking reduction were significant factors.
Barolia et al, 2021^29^	Karachi, Pakistan.	Attitudes and factors influencing cigarette smoking cessation among adult smokers with cardiovascular or respiratory diseases.	Adult smokers visiting outpatient cardiac and respiratory clinics at a private tertiary care hospital in Karachi, Pakistan. The study included 13 participants, both male and female, with smoking-related health risks.	Qualitative descriptive exploratory design, utilizing in-depth, semi-structured interviews.	The study revealed various misconceptions about smoking, the impact of mindset on smoking cessation, and the influence of socio-cultural and institutional factors on smoking behavior. It highlighted the need for tailored smoking cessation strategies that address these misconceptions and factors.
Agyei-Baffour et al, 2020^30^	Kumasi, Ashanti Region, Ghana.	Attitudes and practices regarding Human Papillomavirus (HPV) vaccination among healthcare providers.	Healthcare providers in Kumasi, Ghana, specifically at the Komfo Anokye Teaching Hospital and Ghana Health Services. The study included 29 participants, comprised of physicians, nurses, immunization field officers, and other healthcare professionals.	Qualitative study using focus group discussions, guided by the MTM of behavior change.	The study identified general knowledge about HPV, gaps in understanding of HPV vaccination, and factors that could increase HPV vaccination in Ghana. Barriers included competing health priorities, lack of data on HPV, and confusion about vaccination eligibility and schedule. The study emphasized the need for comprehensive HPV vaccination education and intervention programs in Ghana.
Su et al, 2023^31^	The study was conducted in China, involving participants from Eastern, Central, and Western regions of the country.	The study focused on understanding the factors influencing vaccination intentions, particularly the willingness to receive the COVID-19 vaccine.	The study targeted young adults in China, aged between 18 and 35 years. A total of 12 participants were involved, with a balanced gender ratio and diverse regional representation.	Qualitative research using semi-structured interviews, supplemented by thematic analysis and topic modeling.	The study identified ten key factors influencing COVID-19 vaccination intention among young adults in China. These include the effectiveness and safety of vaccines, the side effects of the vaccine, application range of the vaccine, external influences, direct and transparent information, positive perceptions of the epidemic situation, safe and reliable vaccine-related information, social responsibility, perceived threats to the freedom of choosing vaccination, and policy requirements.
Mousali et al, 2020^32^	Hamadan City, Iran.	Predictors of relapse to drug use among male addicts.	Male addicts referred to addiction treatment centers in Hamadan City, Iran in 2019. The study included 17 participants.	Qualitative research using directed content analysis.	The study identified emotional transformation, changes in the social environment, and changes in the physical environment as the main predictors of drug use relapse. Key factors included unpleasant emotions, peer pressure, and access to drugs.
Dai et al, 2023^33^	University of Nevada, Las Vegas, NV, USA.	Yoga behaviors among college students, based on the MTM of health behavior change.	College students (n = 79, predominantly female) from a health and fitness course centered on Vinyasa Yoga and Mindfulness Meditation.	Qualitative study using comprehensive yoga journaling and directed content analysis.	The study revealed benefits of yoga, such as improved physical performance, reduced stress, acceptance of oneself, and better well-being. Challenges included time commitment and lack of motivation. The study highlights the importance of social support and sustained commitment for maintaining yoga practice.

**Table 3:** Experimental studies utilizing multi-theory model of health behavior change from 2016 to December 2023

**Author & Publication year**	**Location of Study**	**Type of behavior studied**	**Target group (setting)**	**Type of study/design**	**Main Findings**
Brown et al, 202^8^	Greater Jackson area, Mississippi, USA.	Increasing fruit and vegetable consumption among African American women.	African American women, primarily from the Greater Jackson area. The study involved 60 participants, randomly assigned to either the experimental (SAVOR intervention) or comparison (knowledge-based intervention) group.	An RCT with measurements taken at pretest, posttest, and follow-up (eight weeks).	Overall fruit and vegetable consumption: The mean consumption of fruits and vegetables in the experimental group increased significantly from pre-test (2.78) to posttest (4.77) and remained high at follow-up (5.04), compared to the comparison group. Effect Size: measured by partial eta squared, was 0.193. *P* value: < 0.0001​​. Fruit consumption: There was a significant increase in the mean consumption of fruits in the experimental group from pre-test (1.15) to posttest (2.35) and follow-up (2.12). Effect Size: 0.174. *P* value: < 0.0001​​. Vegetable consumption: The mean consumption of vegetables in the experimental group increased significantly from pre-test (1.63) to posttest (2.42) and follow-up (2.92), while the comparison group showed minimal changes. Effect size: 0.072. *P *Value: 0.02. MTM constructs: Statistically significant changes (*P* < 0.0001) were noted for all MTM constructs except for participatory dialogue.​​
Bashirian et al, 2019^9^	Hamadan city, Western Iran.	The research focused on reducing WPS among male adolescent students.	The study involved 94 male adolescent students, divided into an intervention group and a control group, each comprising 47 students.	An RCT with two groups, conducted in 2018, and involving educational intervention sessions.	Participatory dialogue, behavioral confidence, emotional transformation, and practice for change: Significant improvements in these MTM constructs were noted in the intervention group compared to the control group post-intervention (*P* < 0.001 for most constructs). Frequency of WPS: There was a significant reduction in the frequency of WPS in the intervention group, with a decrease from 14.9% to 4.3% compared to the control group (*P <*0.001). These results suggest the effectiveness of the MTM-based educational intervention in reducing WPS among male adolescent students.
Hayes et al, 2019^23^	Central Mississippi, United States.	Initiating and sustaining physical activity among African American women.	African American or African ancestry women, ages 18-69, in Central Mississippi. The study included 50 participants, with 25 in the experimental group and 23 in the comparison group.	Randomized-controlled trial with a pre-test, post-test, and 6-week follow-up evaluation, consisting of two groups: experimental and comparison.	The MTM-based intervention led to significant outcomes: Physical Activity Minutes: Increased from a mean of 37 minutes pre-test to 172 minutes post-test (mean difference 135.08 minutes, *P* < 0.0001). Waist circumference: Reduced from a mean of 39 inches pre-test to 38 inches post-test (mean difference -1.12 inches, *P* < 0.001). Changes in physical environment: Improved from a mean of 7 units pre-test to 9 units post-test (mean difference 2.08 units, *P* < 0.004). These results indicate the efficacy of the MTM-based intervention in improving physical activity and health indicators among African American women.
Yoshany et al, 2021^24^	Yazd, Iran	The effect of specific educational interventions on the quality of life among postmenopausal women.	The study involved 80 postmenopausal women, with 40 participants assigned to each arm of the RCT.	The study design is an RCT with multi-stage stratified random sampling and random allocation to control or intervention group.	Quality of life: The study demonstrated a significant enhancement in the quality of life immediately after the intervention and sustained at 3 months post-intervention in the group that received the intervention (*P* < 0.001 for both time points). Initially, there was no difference between the control and intervention groups regarding quality of life, indicating that the observed improvements were due to the intervention​​. Quality of life domains: Significant improvements were observed in the vasomotor, psychosocial, physical, and sexual domains of the participants' quality of life after receiving the intervention, with all domains showing statistically significant differences favoring the intervention group when compared to the control group (*P* < 0.001)​​.
Joveini et al, 2023^25^	Joven city, Khorasan Razavi province, Iran.	Reduction of sugar consumption to decrease BMI and abdominal obesity in women.	Iranian women aged 30–60 years. The study had 63 participants in the control group and 65 in the intervention group.	Quasi-experimental study with a control and an intervention group, involving educational sessions based on the MTM.	BMI reduction: Six months after the intervention, there was a significant reduction in BMI in the intervention group (average BMI 32.18 ± 4.16) compared to the control group (average BMI 34.15 ± 2.03). The difference was statistically significant (*P* < 0.001). Waist Size Reduction: There was a notable decrease in waist size in the intervention group compared to the control group. Three months post-intervention, the intervention group's average waist size was 96.87 ± 7.36 cm, compared to 100.14 ± 9.31 cm in the control group (*P* < 0.001). This trend continued at six months post-intervention, with the intervention group's waist size further reducing to 95.76 ± 6.84 cm. Overall Impact on MTM Constructs: The study found significant differences in all MTM constructs between the intervention and control groups over time (Time effect), between the groups (Group effect), and in the change over time between the two groups (Time*Group effect), with all *P* values less than 0.05.
Gupta et at, 2023^26^	Chandigarh, a union territory in the northern part of India.	The focus was on portion size consumption behavior and the nutritional status among college students.	The study targeted college students aged 18 to 21 years. The participants were chosen from two private co-educational colleges in Chandigarh with a similar teaching profile.	The research design was quasi-experimental	Reduction in large portion-sized meal consumption: There was a significant decrease in the proportion of participants consuming large portion-sized meals in the intervention group compared to the control group, with 46% in the intervention group versus 11% in the control group showing this reduction (*P *< 0.001)​​​​. Change in meal consumption for breakfast and dinner: The study observed a statistically significant difference in the proportion of participants changing their meal consumption for breakfast (*P* = 0.01) and dinner (*P* = 0.001) between the intervention and control groups​​. Variance in initiation and sustenance of small portion size consumption behavior: About 20.2% of the variance in the initiation of small portion size consumption behavior was explained by changes in the physical environment (*P* = 0.003). Additionally, approximately 49.1% of the variance in the sustenance of this behavior was accounted for by emotional transformation, practice for change, and changes in the social environment (*P* < 0.05)​​. Reduction in BMI and waist-hip ratio: The intervention led to a significant reduction in BMI, from 23.37 kg/m^2^ in the intervention group to 24.14 kg/m^2^ in the control group (difference of means = 0.21, *P* < 0.001). Similarly, there was a reduction in the waist-hip ratio, from 0.86 in the intervention group to 0.92 in the control group (difference of means = 0.02, *P* = 0.007)​.
Kumar et al, 2021^27^	Bangalore, India.	The research focused on tobacco cessation among users visiting a dental college.	The study involved 100 tobacco users, both smokers and smokeless tobacco users.	Non-randomized uncontrolled trial.	Behavioral change across time periods: Significant behavioral changes were observed in the study at different time intervals. There was a marked change from the start of the study to 2 weeks (*P*< 0.001), from the start to 6 weeks (*P*= 0.002), and from the start to 12 weeks (*P*= 0.04). Impact of addiction level on behavioral change: The study found significant behavioral changes depending on the level of tobacco addiction. For those with varying addiction levels, notable changes were seen at 2 weeks (*P*= 0.03) and 6 weeks (*P*= 0.02). Comparison of behavior initiation and sustenance: The research showed that the scores for initiating behavior were significantly higher than those for sustaining behavior. This was observed at the beginning of the study (*P*= 0.016), at 2 weeks (*P*= 0.02), and at 6 weeks (*P*= 0.03).

Abbreviations: WPS, water pipe smoking; RCT, randomized controlled trial; BMI, body mass index; MTM, multi-theory model.

###  Risk of bias assessment

 The risk of bias in the experimental studies, as detailed in Table 1, was meticulously evaluated to ensure the integrity and reliability of the findings related to the effectiveness of the MTM in health behavior change interventions. The assessment utilized a comprehensive approach tailored to the design of each study, employing the Cochrane risk-of-bias tool for randomized trials (RoB 2) for RCTs and the Cochrane risk-of-bias tool for non-randomized studies of interventions (ROBINS-I) for quasi-experimental and non-randomized control trial studies.^21,22^

 The results of the bias assessment revealed that the majority of the RCTs, including those conducted by Hayes et al^23^ and Yoshany et al^24^, demonstrated a low to moderate risk of bias across most domains. Specific areas of concern highlighted in a few studies were related to the randomization process and the selection of reported results, indicating some concerns but not significantly detracting from the overall findings of the studies.

 In the quasi-experimental and non-randomized studies, the risk of bias was generally assessed as moderate. While these studies showed strength in addressing potential confounding factors and ensuring accurate outcome measurement, the inherent limitations of their study designs posed challenges in completely eliminating bias. Nonetheless, the moderate risk does not undermine the valuable insights these studies contribute to understanding the MTM’s application in health behavior change.

 The rigorous assessment of bias underscores the methodological robustness of the included experimental studies, providing a solid foundation for interpreting the effectiveness of the MTM-based interventions. While acknowledging the potential for bias, the comprehensive evaluation process ensures that the findings presented from these experimental studies offer reliable and actionable insights into the MTM’s utility in promoting health behavior change across various contexts.

## Discussion

 The primary aim of this study was to synthesize collective evidence regarding the effectiveness and predictability of MTM as a framework for health behavior change, as demonstrated in peer-reviewed journal articles utilizing qualitative, cross-sectional, and experimental research designs. In the realm of qualitative research on MTM, the predominant data collection methods included interviews and focus group discussions, aligning with common qualitative research practices.^89^ In the majority of these studies, researchers opted for directed content analysis as their chosen approach for data analysis.^28,30,32,33^ Directed content analysis is a well-established method used in studies that adopt a pre-existing theoretical paradigm.^90-93^ This qualitative approach proves invaluable for researchers in the development of MTM-based instruments, drawing insights from community-based samples. Directed content analysis is frequently indispensable in this context. However, the remaining qualitative studies employed conventional thematic analysis,^29,31^ typically associated with grounded theory, to analyze raw data. While thematic analysis has the potential to uncover new constructs that may complement MTM, these two studies did not identify such novel constructs. Future research may explore this avenue further, aiming to identify constructs beyond those proposed by MTM. Some qualitative studies also utilized their data to formulate interventions, representing a crucial application that practitioners and researchers should undertake in the future. Nevertheless, it is essential to acknowledge that qualitative evidence is not generalizable and primarily serves to complement other data sources in comprehending the effectiveness of MTM-based studies.

 The cross-sectional studies, while not providing definitive evidence, play a vital role in establishing the credibility of MTM. Among the 56 cross-sectional studies, 50 studies have assessed the explanatory potential of MTM constructs using hierarchical multiple regression. Both the initiation and sustenance models, examined in 49 and 45 studies respectively, showed similar average explanatory powers (38.4% for initiation and 38.9% for sustenance) with a wide range of effectiveness. Notably, a significant number of studies for both models reported results in the 30% range, indicating a consistent pattern of explanatory potential across the research. These findings underscore the robust predictive power of MTM, especially considering its ability to predict both components of behavior change.^94^ In contrast, other theories such as social cognitive theory,^95^ the theory of planned behavior,^96^ integrative models of behavioral prediction, or reasoned action approaches primarily focus on behavior acquisition rather than behavior change.^97^ MTM’s strength lies in its capacity to facilitate change in behaviors that are typically challenging to modify, aligning with the goals of public health educators and researchers. Furthermore, MTM has been successfully applied to a wide range of behaviors across various target populations. There is a growing need to extend the application of this framework to encompass all behaviors used in health promotion efforts and to examine its effectiveness across diverse global populations. While many interventions have been conducted in the United States and have demonstrated strong predictive power, there is a call for more global applications. Promising global studies include those conducted in Ghana,^34,38^Nigeria,^47^ Fiji,^55^China,^56^Iran,^58^ Nepal,^59^and several studies in India.^39,68,86^ However, it is essential to investigate cases with low explanatory potential, such as two studies from India,^53,71^ to discern the specific factors contributing to the lower explanatory potential. These factors could encompass the chosen behavior, the target population, instrument adaptation/translation, data collection methods, or other variables. Cross-sectional studies, being relatively straightforward to conduct, can serve as the foundation for accumulating further corroborative evidence to support and refine this fourth generation MTM framework. Additionally, cross-sectional studies have significantly contributed to the development and refinement of instrumentation. Most studies have employed expert panels for face and content validation, while construct validation has involved methods such as exploratory factor analysis extensions and structural equation modeling. Both validation methods are robust and can be adopted by future researchers.^94^

 Finally, experimental studies, known for providing the most definitive evidence in research,^89^ have yielded valuable insights into the MTM. Among the seven identified studies, two employed a quasi-experimental design, four utilized the RCT design, and one used a non-randomized uncontrolled trial study design. All seven studies reported positive improvements in the behaviors they aimed to modify, bolstering the potential robustness of the MTM framework. It is worth noting that not all studies provided data on effect sizes, which is a consideration for future intervention researchers. However, the evidence remains limited due to the relatively small number of studies conducted thus far. There is a clear need for more research encompassing a variety of behaviors to thoroughly test the efficacy of the MTM. Subsequently, effectiveness trials in implementation science should be undertaken to assess the real-world impact of MTM-based interventions. Many of the interventions to date have utilized knowledge-based interventions as a comparison group. Future studies may explore comparisons between the MTM and other contemporary theories, expanding the scope of research in this area.

## Implications for practice

 MTM emerges as a valuable model with untapped potential for practical application in the development of potent interventions. To enhance its effectiveness, certain constructs within the initiation model can be further refined. For instance, the construct of participatory dialogue can be reinforced by emphasizing the advantages of behavior change while addressing potential disadvantages in interventions. Similarly, the construct of behavioral confidence can be strengthened by breaking down behavior change into manageable steps and exploring various sources of support to bolster confidence. The changes in the physical environment construct can be operationalized by providing tangible resources or implementing policies that facilitate and support behavior change. By fine-tuning these aspects, MTM can become an even more effective tool for promoting positive health behavior change.

 Refining constructs within the sustenance component of MTM can further enhance the behavior change outcome. To modify the construct of emotional transformation, efforts should focus on identifying and channeling emotions toward the goals of behavior change, helping individuals develop a more positive and constructive emotional relationship with the desired behaviors. Similarly, the construct of practice for change should encourage individuals to maintain constant awareness of the behavior change process, facilitating ongoing evaluation and adjustment of strategies to ensure continued success. Lastly, changes in the social environment should be fostered through robust support systems that encompass family, friends, health professionals, social media, and other societal venues.

 There is a pressing need to develop behavior change interventions based on MTM in a wide range of settings, including schools, community settings, worksites, universities/colleges, and other locations. Health education specialists working in these settings should consider employing the MTM framework to address a diverse array of health behaviors among various target populations. By leveraging MTM, they can design tailored interventions that are effective in promoting positive health behavior changes in their respective communities and organizations.

## Recommendations for research

 Researchers should prioritize the continued refinement of instrumentation in MTM-based studies. Qualitative research can play a valuable role in identifying additional constructs that may enhance the model’s comprehensiveness. For instance, in the initiation model, future research could explore the potential role of changes in the social environment as an adjunct, aligning with the perspectives of many social psychologists. Additionally, test-retest reliability, which has been largely overlooked in previous studies, should be a focus for future research. Establishing the stability of instruments, particularly in the context of evaluating intervention efficacy, is essential to ensure the reliability of findings. By addressing these aspects, researchers can contribute to the ongoing development and robustness of MTM as a valuable framework for health behavior change.

 Future research efforts should encompass both cross-sectional studies aimed at identifying the determinants of behavior change and experimental studies designed for efficacy testing using MTM. In the case of efficacy testing, RCTs should be the preferred method. However, for specific subgroups like school children, variations such as group RCTs may be more suitable and applicable. These research endeavors will contribute to a deeper understanding of the effectiveness and applicability of MTM in diverse settings and among various target populations, ultimately advancing the field of health behavior change.

## Limitations

 This review had several limitations that warrant consideration. First, the use of a general search term for MTM without specific health behavior-related terms may have resulted in the omission of studies focused on particular health behaviors. Consequently, some relevant research may not have been included. To mitigate this limitation, future research could adopt a more targeted approach by incorporating specific behavior-related keywords into the search strategy. Second, the substantial heterogeneity in the health behaviors examined across studies posed challenges for conducting a meaningful quantitative analysis or comparison of findings. To address this issue, researchers may consider conducting separate systematic reviews or meta-analyses for distinct health behaviors or grouping studies by behavior type. Third, the reliance on only three databases for article retrieval might have overlooked pertinent studies, indicating a potential limitation. To mitigate this limitation, researchers can broaden their search scope by including additional reputable databases. Fourth, because of the heterogeneity of behaviors, a meta-analysis could not be performed. Finally, there is a possibility of publication bias, as smaller-scale or less statistically powered studies might not have been included in this review. To address potential publication bias, future research can incorporate strategies like thorough grey literature searches and the inclusion of studies with smaller sample sizes to provide a more balanced representation of available evidence. Lastly, due to the exclusion of the non-English studies, generalizability will be limited. This underscores the importance of broadening the search to include other languages in future studies.

## Conclusion

 This systematic review aimed to evaluate existing research employing the MTM framework to forecast and foster the initiation and sustenance of diverse health behavior changes. Thus far, this model has been employed in various behavioral contexts and environments. Findings from these studies underscore the potential usefulness of MTM in elucidating and promoting health behavior change, thanks to its adaptable constructs. The application of MTM as a theoretical framework should persist in future investigations dedicated to instigating enduring and beneficial health behavior transformations.

## Acknowledgements

 We would like to thank the Department of Social and Behavioral Health, Department of Internal Medicine, and the School of Public Health at the University of Nevada at Las Vegas (UNLV) and various institutions that carried out research on MTM for their support. We also thank the numerous participants who participated in the research.

## Competing Interests

 Manoj Sharma is the originator of the MTM.

## Ethical Approval

 Not applicable.

## Supplementary Files


Supplementary file 1 contains Tables S1-S3 and an example of the search strategy for the PubMed database.
