# Supplementary material for: Utilization of the multi-theory model (MTM) of health behavior change to explain health behaviors: A systematic review
Source: Health Promot Perspect. 2024 Jul 29;14(2):121–35. doi: 10.34172/hpp.42887 (PMC11403345; doi:10.34172/hpp.42887)
Supplement: Supplementary file 1 — contains Tables S1-S3 and an example of the search strategy for the PubMed database. [file hpp-14-121-s001.pdf]

Kapukotuwa et al, **Health Promotion Perspectives**, 2024, 14(2), 121-S1
doi: 10.34172/hpp.42887
https://hpp.tbzmed.ac.ir

Supplementary File 1. An Example of Search Strategy for the PubMed Database

((("MTM"[Title/Abstract] OR "multi theory model"[Title/Abstract] OR "multi theory model"[Title/Abstract] OR "multi theory model"[Title/Abstract] OR ("initiation"[Title/Abstract] OR "sustenance"[Title/Abstract])) AND ("health behavior"[MeSH Terms] OR (("obesogen"[All Fields] OR "obesogenic"[All Fields] OR "obesogenicity"[All Fields] OR "obesogens"[All Fields]) AND "behavior"[MeSH Terms]) OR (("human s"[All Fields] OR "humans"[MeSH Terms] OR "humans"[All Fields] OR "human"[All Fields]) AND "behavior"[MeSH Terms]))) NOT ("review"[Publication Type] OR "systematic review"[Publication Type])) AND ((fft[Filter]) AND (humans[Filter]) AND (2016/1/1:2023/12/31[pdat]) AND (english[Filter]))

Supplementary Table 1: Preferred Reporting Items for Systematic reviews and Meta-Analyses checklist

Section and Topic	Item #	Checklist item	Location where item is reported
**TITLE**			
Title	1	Identify the report as a systematic review.	Page 1
**ABSTRACT**			
Abstract	2	See the PRISMA 2020 for Abstracts checklist.	Pages 1
**INTRODUCTION**			
Rationale	3	Describe the rationale for the review in the context of existing knowledge.	Page 4
Objectives	4	Provide an explicit statement of the objective(s) or question(s) the review addresses.	Page 4
**METHODS**			
Eligibility criteria	5	Specify the inclusion and exclusion criteria for the review and how studies were grouped for the syntheses.	Pages 5
Information sources	6	Specify all databases, registers, websites, organisations, reference lists and other sources searched or consulted to identify studies. Specify the date when each source was last searched or consulted.	Page 5
Search strategy	7	Present the full search strategies for all databases, registers and websites, including any filters and limits used.	Page 5
Selection process	8	Specify the methods used to decide whether a study met the inclusion criteria of the review, including how many reviewers screened each record and each report retrieved, whether they worked independently, and if applicable, details of automation tools used in the process.	Page 5
Data collection process	9	Specify the methods used to collect data from reports, including how many reviewers collected data from each report, whether they worked independently, any processes for obtaining or confirming data from study investigators, and if applicable, details of automation tools used in the process.	Page 6
Data items	10a	List and define all outcomes for which data were sought. Specify whether all results that were compatible with each outcome domain in each study were sought (e.g. for all measures, time points, analyses), and if not, the methods used to decide which results to collect.	Page 6
	10b	List and define all other variables for which data were sought (e.g. participant and intervention characteristics, funding sources). Describe any assumptions made about any missing or unclear information.	Page 6

Section and Topic	Item #	Checklist item	Location where item is reported
Study risk of bias assessment	11	Specify the methods used to assess risk of bias in the included studies, including details of the tool(s) used, how many reviewers assessed each study and whether they worked independently, and if applicable, details of automation tools used in the process.	Page 6
Effect measures	12	Specify for each outcome the effect measure(s) (e.g. risk ratio, mean difference) used in the synthesis or presentation of results.	Page 6
Synthesis methods	13a	Describe the processes used to decide which studies were eligible for each synthesis (e.g. tabulating the study intervention characteristics and comparing against the planned groups for each synthesis (item #5)).	N/A
	13b	Describe any methods required to prepare the data for presentation or synthesis, such as handling of missing summary statistics, or data conversions.	N/A
	13c	Describe any methods used to tabulate or visually display results of individual studies and syntheses.	N/A
	13d	Describe any methods used to synthesize results and provide a rationale for the choice(s). If meta-analysis was performed, describe the model(s), method(s) to identify the presence and extent of statistical heterogeneity, and software package(s) used.	N/A
	13e	Describe any methods used to explore possible causes of heterogeneity among study results (e.g. subgroup analysis, meta-regression).	N/A
	13f	Describe any sensitivity analyses conducted to assess robustness of the synthesized results.	N/A
Reporting bias assessment	14	Describe any methods used to assess risk of bias due to missing results in a synthesis (arising from reporting biases).	N/A
Certainty assessment	15	Describe any methods used to assess certainty (or confidence) in the body of evidence for an outcome.	N/A
**RESULTS**			
Study selection	16a	Describe the results of the search and selection process, from the number of records identified in the search to the number of studies included in the review, ideally using a flow diagram.	Figure 1
	16b	Cite studies that might appear to meet the inclusion criteria, but which were excluded, and explain why they were excluded.	Supplementary Table 1
Study characteristics	17	Cite each included study and present its characteristics.	Pages 7-9<br>Tables 3-5
Risk of bias in studies	18	Present assessments of risk of bias for each included study.	Table 2
Results of individual studies	19	For all outcomes, present, for each study: (a) summary statistics for each group (where appropriate) and (b) an effect estimates and its precision (e.g. confidence/credible interval), ideally using structured tables or plots.	Tables 3-5
Results of syntheses	20a	For each synthesis, briefly summarise the characteristics and risk of bias among contributing studies.	N/A
	20b	Present results of all statistical syntheses conducted. If meta-analysis was done, present for each the summary estimate and its precision (e.g. confidence/credible interval) and measures of statistical heterogeneity. If comparing groups, describe the direction of the effect.	N/A
	20c	Present results of all investigations of possible causes of heterogeneity among study results.	N/A
	20d	Present results of all sensitivity analyses conducted to assess the robustness of the synthesized results.	N/A
Reporting biases	21	Present assessments of risk of bias due to missing results (arising from reporting biases) for each synthesis assessed.	N/A
Certainty of evidence	22	Present assessments of certainty (or confidence) in the body of evidence for each outcome assessed.	N/A
**DISCUSSION**			

Section and Topic	Item #	Checklist item	Location where item is reported
Discussion	23a	Provide a general interpretation of the results in the context of other evidence.	Pages 10-13
	23b	Discuss any limitations of the evidence included in the review.	Pages 14-15
	23c	Discuss any limitations of the review processes used.	Pages 14-15
	23d	Discuss implications of the results for practice, policy, and future research.	Page 12-14
**OTHER INFORMATION**			
Registration and protocol	24a	Provide registration information for the review, including register name and registration number, or state that the review was not registered.	N/A
	24b	Indicate where the review protocol can be accessed, or state that a protocol was not prepared.	N/A
	24c	Describe and explain any amendments to information provided at registration or in the protocol.	N/A
Support	25	Describe sources of financial or non-financial support for the review, and the role of the funders or sponsors in the review.	N/A
Competing interests	26	Declare any competing interests of review authors.	MS is the originator of MTM
Availability of data, code and other materials	27	Report which of the following are publicly available and where they can be found: template data collection forms; data extracted from included studies; data used for all analyses; analytic code; any other materials used in the review.	From SK

Supplementary Table 2. Studies that met the inclusion criteria but excluded from the review

Author & Publication year	Study Title	Reason for Exclusion
(Sharma, M., 2016)[12]	A new theory for health behavior change: Implications for alcohol and drug education.	Editorial
(Sharma, M., 2017)[13]	Trends and prospects in public health education: A commentary.	Editorial
(Ghaffarifar & Ghouchani, 2017)[14]	Health educators' role in health promotion from a new perspective: A new theory in their professional toolbox.	Editorial
(Sharma & Sharma, 2019)[15]	Algorithm-driven fourth generation multi-theory model for alcohol and drug education.	Editorial
(Sharma & Nahar, 2017)[16]	New approach for promoting HPV vaccination in college men based on multi-theory model (MTM) of health behavior change.	Letter to the Editor
(Kim et al., 2018)[17]	Influencing health behavior change related to musculoskeletal conditions: The need to expand the evidence base and the case for multitheory model.	Letter to the Editor
(Morowatisharifabad et al., 2019)[18]	Effects of an educational intervention based on the multi-theory model on promoting the quality of life in postmenopausal women: A protocol.	Protocol
(Sharma, M., 2021)[19]	A protocol for assessing the readiness for practicing meditation (manan dhyana) as a tool for reduction of stress among high-risk occupations.	Protocol
(Jiang et al., 2023)[20]	Impact of a mobile health intervention based on multi-theory model of health behavior change (MTM) on self-management in patients with differentiated thyroid cancer: Protocol for a randomized control trial.	Protocol

Supplementary Table 3: Descriptive and cross-sectional studies utilizing Multi-Theory Model (MTM) of health behavior change from 2016 to December 2023.

Author & Publication year	Location of Study	Type of behavior studied	Target group (setting)	Type of study/design	Main Findings
(Sharma et al., 2021c)[7]	Southern USA University.	COVID-19 vaccine acceptance among college students.	282 participants, majority female (62.8%) and White (56.4%).	Cross-sectional study using a 27-item questionnaire.	Initiation of COVID-19 Vaccine Acceptance: Among college students, the significant predictors for initiating COVID-19 vaccine acceptance were behavioral confidence ($p < 0.001$), participatory dialogue ($p < 0.001$), and changes in the physical environment ($p = 0.001$), accounting for 54.8% of the variance. For those hesitant about the vaccine, behavioral confidence ($p < 0.001$) and Republican Party affiliation ($p = 0.004$, negatively associated) were significant, explaining 60.6% of the variance. Sustenance of COVID-19 Vaccine Acceptance: The study focused primarily on the initiation of vaccine acceptance and did not specifically address the sustenance aspect.
(Asare et al., 2020)[34]	Ashanti Region, Ghana.	HPV vaccination initiation and completion among adolescents.	Adolescents aged 12-17 years from four schools, 285 participants.	Cross-sectional study.	Initiation of HPV Vaccination: The final model for initiation found perceived beliefs and change in physical environment as significant predictors. Perceived beliefs had a p-value of <0.001 and change

					in physical environment also had a p-value of <0.001, accounting for a combined variance of 15%. Sustenance of HPV Vaccination: The final model for sustenance identified perceived beliefs, practice for change, and emotional transformation as significant predictors, each with a p-value of <0.001. These factors explained a total variance of 16.9% in sustaining the HPV vaccination behavior.
(Sarwar et al., 2021)[35]	A university in the United States.	COVID-19 vaccine acceptance among college students.	55 undergraduate students, majority female, younger, Hispanic or Latino.	Pilot study with a survey conducted in summer 2021.	The study explored factors affecting students' vaccine acceptance, focusing on mistrust, misinformation, religious beliefs, and ethical implications. It emphasized the importance of addressing these factors to increase vaccine uptake among college students.
(Sharma et al., 2021f)[36]	United States.	COVID-19 vaccine hesitancy among African Americans.	428 unvaccinated African Americans.	Cross-sectional study using a web-based survey and a 28-item questionnaire.	Initiation of COVID-19 Vaccine Acceptance Among African Americans: The study identified participatory dialogue and behavioral confidence as significant predictors for initiating COVID-19 vaccine acceptance among African Americans. The model explained 65% of the variance in vaccine acceptance, with

					participatory dialogue and behavioral confidence having a p-value < 0.001. Sustenance of COVID-19 Vaccine Acceptance: The study did not specifically address the sustenance of COVID-19 vaccine acceptance, focusing primarily on the initiation of vaccine acceptance among African Americans.
(Batra et al., 2022a)[37]	United States.	COVID-19 booster vaccination hesitancy.	Nationally representative sample of U.S. adults.	Cross-sectional study using a 52-item psychometric valid web-based survey.	Initiation of COVID-19 Booster Vaccination: In the final model of the study, for the initiation of COVID-19 booster vaccination, behavioral confidence and changes in the physical environment were significant predictors. Behavioral confidence had a p-value < 0.001, and changes in the physical environment had a p-value < 0.05. The model accounted for 62.9% of the variance. Sustenance of COVID-19 Booster Vaccination: The study did not specifically address sustenance in the context of booster vaccination. It focused more on the factors influencing the initiation or acceptance of the COVID-19 booster vaccine.
(Popelsky et al., 2022)[38]	Ashanti Region, Ghana.	HPV vaccination among parents of unvaccinated adolescents.	380 parents, predominantly female (91.1%).	Cross-sectional study using a 44-item validated survey.	Initiation of HPV Vaccination: The final model for the initiation of HPV vaccination among

					Ghanaian parents showed that changes in the physical environment and behavioral confidence were significant predictors. Changes in the physical environment had a p-value < 0.05, and behavioral confidence had a p-value < 0.001. The variance explained by this model was 13.6%. Sustenance of HPV Vaccination: The final model for the sustenance of HPV vaccination behavior highlighted emotional transformation and changes in the social environment as significant predictors, both with p-values < 0.001. The variance explained by these predictors was 45.8%.
(Achrekar et al., 2022)[39]	India.	COVID-19 booster dose hesitancy.	Indian residents.	Cross-sectional study with 687 participants.	Initiation of COVID-19 Booster Vaccination: In the final model, participatory dialogue and changes in the physical environment had p-values < 0.001, while behavioral confidence had a p-value < 0.05. The variance explained was 51.4%. The study did not include an analysis of the sustenance of COVID-19 booster vaccination.
(Batra et al., 2022b)[40]	United States.	COVID-19 vaccination hesitancy among parents for their children.	The study assessed a national random sample of 263 parents. Type of	Cross-sectional study.	The study found that 42% of parents were not willing to vaccinate their children for COVID-19. Parental

			Study/Design: Cross-sectional study.		vaccination status, booster dose acceptance, education, and political affiliation were significant predictors of willingness to vaccinate children. Behavioral confidence and participatory dialogue were key predictors of COVID-19 vaccination hesitancy among parents.
(Nerida et al., 2023)[41]	Nevada, USA.	Behavior of COVID-19 vaccine acceptance among Hispanics/Latinxs.	The study involved 231 Hispanic/Latinx individuals, predominantly of Mexican descent, from various educational and income levels.	The research was a quantitative, cross-sectional, survey-based study.	Initiation of COVID-19 Vaccine Acceptance: The model explained 63.0% of the variability in the initiation of COVID-19 vaccine acceptance among vaccine-hesitant individuals. Participatory dialogue and behavioral confidence were the significant constructs, both displaying a p-value of <0.001. Sustenance of COVID-19 Vaccine Acceptance: For sustenance among vaccine-hesitant individuals, the model accounted for 37.4% of the variability. Emotional transformation was the significant construct with a p-value of <0.001.
(Sharma et al., 2016)[42]	A large Southern public university in the United States.	Initiation and sustenance of small portion size consumption among college students.	College students, with a total of 135 participants. The study focused on those who consumed large portion sizes within the past 24 hours.	Cross-sectional study using a survey based on the Multi-Theory Model (MTM) of health behavior change.	The initiation of small portion size consumption, which explained 37% of the variance, was significantly influenced by participatory dialogue (advantages outweighing

					disadvantages) with a p-value of 0.006, behavioral confidence with a p-value of <0.001, age with a p-value of 0.005, and gender, where males were less likely than females to initiate this behavior, with a p-value of 0.008. These findings suggest that personal beliefs, confidence, and demographic factors like age and gender play a vital role in starting healthier eating habits. Regarding the sustenance of small portion size consumption, accounting for 20.5% of the variance, the study identified emotional transformation as a significant predictor with a p-value of 0.001, changes in the social environment with a p-value of 0.033, and racial differences, where Whites were less likely than other races to sustain this behavior, with a p-value of 0.001. This indicates the importance of emotional and social factors, as well as racial considerations, in maintaining changes in eating behavior over time.
(Sharma et al., 2018a)[43]	A large public university in the Southeastern United States.	Initiation and sustenance of fruit and vegetable consumption among college students.	College students at the university, with 175 participants who were not consuming the recommended	Cross-sectional study using a survey based on the Multi-Theory Model (MTM) of health	Initiation of Fruit and Vegetable Consumption: In the final model for initiation, the significant predictors were behavioral

			number of fruits and vegetables.	behavior change.	confidence and changes in the physical environment. Behavioral confidence had a p-value of <.001, and changes in the physical environment also had a significant p-value of .001, explaining 40.2% of the variance in the initiation of fruit and vegetable consumption. Sustenance of Fruit and Vegetable Consumption: For the sustenance of this behavior, the final model identified emotional transformation, practice for change, and changes in the social environment as significant predictors. Emotional transformation had a p-value of .030, practice for change had a p-value of <.001, and changes in the social environment had a p-value of .025, collectively accounting for 30.4% of the variance in sustaining the behavior change.
(Lakhan et al., 2019)[44]	Rural Appalachia, USA.	Initiation and sustenance of small portion size consumption behavior.	Residents of six rural Appalachian counties in Kentucky, 156 participants.	Cross-sectional study.	Initiation of Small Portion Size Consumption: In the final model for the initiation of small portion size consumption, significant predictors were participatory dialogue (advantages outweighing disadvantages) with a p-value of 0.033, behavioral

					confidence with a p-value of <0.0001, and changes in the physical environment with a p-value of <0.0001. These factors collectively explained 47.9% of the variance in initiating small portion size consumption. Sustenance of Small Portion Size Consumption: For the sustenance of this behavior, the final model identified emotional transformation and practice for change, both with p-values of <0.0001. These predictors accounted for 40.2% of the variance in sustaining the behavior change.
(Brown et al., 2019)[45]	Mississippi, USA.	Fruit and vegetable consumption among African American women.	African American women from churches in Mississippi, 116 participants.	Cross-sectional study using the Multi-Theory Model (MTM).	Initiation of Fruit and Vegetable Consumption: For the initiation of fruit and vegetable consumption, the final model identified participatory dialogue (advantages outweighing disadvantages) with a p-value of 0.009, behavioral confidence with a p-value of 0.0001, and changes in the physical environment with a p-value of 0.0001. These predictors accounted for 50.8% of the variance in the intention to initiate fruit and vegetable consumption behavior. Sustenance of Fruit and Vegetable

					Consumption: In the final model for the sustenance of fruit and vegetable consumption, significant predictors were emotional transformation (p = 0.0001), practice for change (p = 0.016), and changes in the social environment (p = 0.0001). These factors explained 59.9% of the variance in sustaining the behavior.
(Williams et al., 2020)[46]	Barbershops in Jackson, Mississippi, USA.	Fruit and vegetable consumption among African American men.	134 African American adult men, average age 33.83 years.	Cross-sectional study using a self-administered questionnaire.	Initiation of Fruits and Vegetables Consumption: The study by Williams et al. found that behavioral confidence and changes in the physical environment were significant predictors for the initiation of fruits and vegetables consumption among African American men. These factors accounted for 40.8% of the variance in predicting this behavior change, with behavioral confidence and changes in the physical environment having p-values of <0.0001. Sustenance of Fruits and Vegetables Consumption: For sustaining this behavior, practice for change and emotional transformation were the significant predictors, explaining 37.5% of the variance.

					Practice for change had a p-value of <0.0001, and emotional transformation had a p-value of 0.016.
(Dokun-Mowete et al., 2019)[47]	Nigeria.	Initiation and maintenance of low-salt intake among Nigerian adults with hypertension.	149 Nigerian adults living with hypertension.	Quantitative cross-sectional study.	Initiation of Low-Salt Intake: In the final model for the initiation of low-salt intake, the significant predictors were advantages outweighing disadvantages (p = 0.038), behavioral confidence (p < 0.0001), and changes in the physical environment (p < 0.0001), explaining 40.6% of the variance in the behavior change towards initiating low-salt consumption. Sustenance of Low-Salt Intake: For the sustenance of low-salt intake, the final model identified emotional transformation (p = 0.008), practice for change (p < 0.0001), and changes in the social environment (p < 0.0001) as significant predictors. These factors accounted for 41.8% of the variance in sustaining the consumption of a low-salt diet.
(Xhakollari et al., 2021)[48]	Italy.	Adherence to a Gluten-Free Diet (GFD) among adults with and without celiac disease.	308 participants, majority female (80.19%), average age 39 years.	Cross-sectional study using Integrative Model (IM) and Multi Theory Model (MTM).	Attitudes towards GFD, self-efficacy, and injunctive norms were key factors in adherence. The study found that IM constructs effectively explain adherence to GFD, but MTM constructs for continuation did

					not explain adherence.
(Yoshany et al., 2022)[49]	Yazd, Iran.	Nutritional behaviors in postmenopausal women.	204 postmenopausal women, aged 45–55 years.	Cross-sectional study based on the Multi-Theory Model (MTM).	Initiation of Nutritional Behaviors for Menopausal Symptoms: The study examined predictors in initiating and maintaining nutritional behaviors to address menopausal symptoms. For initiation, significant predictors were participatory dialogue ($p = 0.040$), behavioral confidence ($p < 0.001$), and changes in the physical environment ($p = 0.007$), accounting for a notable variance in initiating these behaviors. Variance was not provided. Sustenance of Nutritional Behaviors: In sustaining these behaviors, emotional transformation was the sole significant predictor, with a p-value of <0.001, indicating its strong influence on the maintenance of nutritional behaviors in menopausal women.
(Nahar et al., 2016)[50]	Southern United States, at a large university.	Initiation and sustenance of physical activity behavior among college students.	College students, with a total of 141 participants who were predominantly female (72.3%) and Caucasian (70.9%). The average age of the participants was approximately 24.56 years.	Cross-sectional study using a survey based on the Multi-Theory Model (MTM) of health behavior change.	For the initiation of physical activity, which explained 26% of the variance, the significant factors included behavioral confidence, changes in the physical environment, and work status, each with p-values of

					<0.001, 0.008, and 0.004, respectively. Moreover, the aspect of advantages outweighing disadvantages was also significant with a p-value of 0.018, highlighting the critical elements that influence the start of physical activity. Regarding the sustenance of physical activity, accounting for 29.7% of the variance, significant contributors were practice for change, emotional transformation, and changes in the social environment. Practice for change demonstrated a substantial impact with a p-value of <0.001. Emotional transformation and changes in the social environment were significant too, with p-values of 0.019 and 0.022, respectively. This emphasizes the importance of ongoing practice, emotional aspects, and social factors in maintaining physical activity over time.
(Hayes et al., 2018)[51]	Jackson, Central Mississippi, USA.	Initiating and sustaining physical activity among African American women.	African American women aged 18 years and older, recruited primarily from churches in Jackson, Mississippi. The study involved 156 participants.	Cross-sectional study.	For the initiation of physical activity, the final regression model identified three significant predictors. Advantages minus disadvantages was a notable factor with a p-value of 0.029. Behavioral confidence also played a crucial role, with a p-value of 0.001, and changes in the physical

					environment were significant with a p-value of 0.004. These variables collectively explained 32.7% of the variance in initiating physical activity. Regarding the sustenance of physical activity, the study found that emotional transformation and changes in the social environment were significant in the final model. Emotional transformation had a p-value of 0.001, and changes in the social environment also had a significant impact, with a p-value of 0.001. Practice for change was not a significant predictor in this final model. Together, these factors accounted for 38.8% of the variance in sustaining physical activity behavior.
(Nahar et al., 2019b)[52]	Southeastern United States, at an osteopathic medical school.	Initiating and sustaining physical activity among osteopathic medical students.	Osteopathic medical students, with 135 participants meeting the study criteria.	Cross-sectional study using an MTM-based survey.	Initiation of Physical Activity: Behavioral confidence was the only significant predictor in the final model for initiation, with a p-value of <.001, accounting for 25.8% of the variance in initiating physical activity behavior. Sustenance of Physical Activity: In the final model for sustenance, significant predictors were changes in social environment (p = .002) and emotional transformation (p <

					.001), explaining 41.7% of the variance in sustaining physical activity behavior.
(Khanna et al., 2020)[53]	District Ambala, Haryana, India.	Physical activity among upper elementary school children.	214 upper elementary school children. The mean age of participants was 10.51 years, with 53.74% boys and 46.26% girls.	Cross-sectional study using a Multi-Theory Model (MTM) based questionnaire.	Initiation of Physical Activity Behavior: The study by Khanna et al. explored the predictors for initiating physical activity among upper elementary school children in Northern India. Behavioral confidence and changes in the physical environment were significant predictors. The model accounted for 12.5% of the variance in the intention to initiate physical activity, with behavioral confidence and changes in the physical environment having p-values of 0.004 and 0.001, respectively. Sustenance of Physical Activity Behavior: For sustaining physical activity behavior, emotional transformation and practice for change were identified as significant predictors. These factors explained 5.3% of the variance in the intention for the sustenance of physical activity, with emotional transformation and practice for change having p-values of 0.012 and 0.008, respectively.
(Yoshany et al., 2022)[54]	Yazd, Iran.	Regular physical activity behavior and quality of life in post-menopausal women.	200 post-menopausal women aged 45-55 years.	Cross-sectional research using the Multi-	Initiation of Regular Physical Activity: The study by Yoshany et al.

				Theory Model (MTM).	focused on regular physical activity behavior in post-menopausal Iranian women. Behavioral confidence and changes in the physical environment were identified as significant predictors for initiating regular physical activity, with p-values of <0.0001 and 0.013, respectively. These predictors accounted for a substantial variance in initiating this behavior. Sustenance of Regular Physical Activity: Emotional transformation and changes in the social environment significantly predicted the sustenance of regular physical activity, with p-values of <0.0001 and 0.017, respectively. These factors were pivotal in maintaining regular physical activity in the target population. Variance information was not provided.
(Shirotriya et al., 2022)[55]	Fiji.	Physical activity behavior among university students.	The study involved 334 students, with 15.6% not engaged in any physical activity (PA). The majority were females (65.9%), undergraduates (93.7%), and Indo-Fijians (68.0%). The average age of participants was 23.70 years.	A cross-sectional study.	Initiation of Physical Activity Behavior: The final model for the initiation of physical activity behavior explained 46.2% of the variance. Changes in the physical environment was the only significant predictor, with a p-value of <0.05. Sustenance of Physical Activity Behavior: The final

					model for the sustenance of physical activity behavior explained 38.8% of the variance. However, none of the constructs in the model were found to be significant.
(Zhang et al., 2022)[56]	Hangzhou, Zhejiang Province, China.	Physical activity behavior among pregnant women.	The study involved 450 pregnant women.	Cross-sectional study.	Initiation of Physical Activity Behavior: The final model for the initiation of physical activity behavior explained 52.1% of the variance. Significant predictors in this model included participatory dialogue ($p = 0.002$), behavioral confidence ($p < 0.001$), changes in physical environment ($p = 0.005$), gestation age ($p = 0.001$), and Gestational Diabetes Mellitus (GDM) ($p < 0.001$). Sustenance of Physical Activity Behavior: The final model for sustenance explained 49.1% of the variance. Significant predictors were emotional transformation ($p < 0.001$), practice for change ($p = 0.001$), changes in social environment ($p < 0.001$), pre-pregnancy exercise habit ($p = 0.001$), and GDM ($p = 0.003$).
(Nahar et al., 2019a)[57]	Rural Appalachian Kentucky, USA.	Smoking cessation intentions among smokers.	Smokers from a shopping mall in rural Appalachian Kentucky, 148 participants.	Cross-sectional study using an MTM-based survey.	Initiation of Smoking Cessation: The final model for initiation identified participatory dialogue ($p = 0.003$) and behavioral confidence (p =

					0.005) as significant predictors, explaining 23.6% of the variance in the behavior change towards initiating smoking cessation. Sustenance of Smoking Cessation: For the sustenance of smoking cessation, emotional transformation was the significant predictor in the final model (p = 0.001), accounting for 23.3% of the variance in sustaining the behavior change.
(Bashirian et al., 2020)[58]	Hamadan city, Western Iran.	Reducing waterpipe smoking in male adolescents.	High school male students (grades 10–12), with 200 participants who smoked waterpipe in the last 30 days.	Cross-sectional study.	Initiation of Reducing Water Pipe Smoking: In the study, participatory dialogue (advantages minus disadvantages) and behavioral confidence were significant predictors for initiating the reduction of water pipe smoking, with p-values of .001 for each. These factors explained 35.7% of the variance in the initiation behavior. Sustenance of Reducing Water Pipe Smoking: For sustaining the behavior, emotional transformation (p = .001), practice for change (p = .002), and changes in the social environment (p = .032) were identified as significant predictors, accounting for 13.5% of the variance in sustaining the reduction of water pipe smoking.

(Sharma et al., 2020d)[59]	Kathmandu Metropolitan City, Nepal.	Smoking cessation.	132 participants, predominantly male (75%), median age 35.53 years.	Cross-sectional study using face-to-face interviews and MTM-based survey.	Initiation of Smoking Cessation: The study found that behavioral confidence and changes in the physical environment were significant predictors for the initiation of smoking cessation, explaining 48% of the variance in this behavior. Behavioral confidence had a p-value of 0.02, while changes in the physical environment had a p-value of 0.01. Sustenance of Smoking Cessation: For the sustenance of smoking cessation, emotional transformation was the significant predictor, accounting for 54% of the variance. The p-value for emotional transformation was less than 0.001.
(Sharma et al., 2022d)[60]	United States.	Vaping quitting behavior among young adults.	The study involved a nationally representative sample of 619 young adults aged 18–24 years who were engaged in vaping behavior.	This was a cross-sectional study using a 49-item web-based survey.	Initiation of Vaping Quitting Behavior: The significant predictors for the initiation of vaping quitting behavior in the final model were participatory dialogue, behavioral confidence, and changes in the physical environment. All these predictors were significant with a p-value of <0.001. The model explained 41.7% of the variance in the initiation of vaping quitting behavior. Sustenance of Vaping Quitting Behavior: For the sustenance of

					vaping quitting behavior, the significant predictors in the final model included emotional transformation ($p < 0.05$), practice for change ($p < 0.001$), and changes in the social environment ($p < 0.001$). The model accounted for 36.6% of the variance in sustaining vaping quitting behavior.
(Nahar et al., 2020)[61]	Southeastern United States, at a private College of Veterinary Medicine.	Initiation and sustenance of stress management behaviors among veterinary students.	Veterinary students who were not already engaging in daily stress management behaviors, totaling 140 participants.	Cross-sectional study.	Initiation of Intentional Relaxation Behavior: For the initiation of intentional relaxation behavior, the final model showed behavioral confidence as the only significant predictor, with a p-value of <0.001, accounting for 49.5% of the variance in the behavior change. Sustenance of Intentional Relaxation Behavior: In the final model for the sustenance of intentional relaxation behavior, emotional transformation was the only significant predictor, with a p-value of <0.001, explaining 50.4% of the variance in sustaining the behavior.
(Sharma et al., 2020c)[62]	Southeastern university in the United States.	Intentional outdoor nature contacts for stress reduction among college students.	281 undergraduate students, with a mean age of 21.69 years, 60% female, 58% White or Caucasian American.	Cross-sectional study using a self-administered questionnaire.	Initiation of Intentional Outdoor Nature Contact: In this study, the initiation of intentional outdoor nature contact among college students was significantly

					predicted by behavioral confidence and changes in the physical environment. These predictors explained 57.5% of the variance in initiating this behavior, with both factors having p-values of less than 0.001. Sustenance of Intentional Outdoor Nature Contact: For the sustenance of this behavior, emotional transformation, practice for change, and changes in the social environment were identified as significant predictors. Together, they accounted for 31.0% of the variance in sustaining the behavior. Emotional transformation had a p-value of 0.021, practice for change had a p-value of less than 0.001, and changes in the social environment had a p-value of 0.002.
(Sharma et al., 2022b)[63]	Southern United States.	Introspective meditation for stress reduction among college students.	65 college students, 75.4% female, 64.6% White, mean age 27.72 years.	Cross-sectional pilot study using a 52-item web-based survey.	Initiation of Introspective Meditation: The final model for initiation found changes in the physical environment as a significant predictor. This variable was statistically significant, with a p-value of <0.05. The model explained a variance of 21.1% in initiating

					introspective meditation among participants. Sustenance of Introspective Meditation: The final model for sustenance identified emotional transformation as a significant predictor. This factor was statistically significant, with a p-value of <0.001. The model explained a total variance of 50.5% in sustaining introspective meditation behavior among the participants.
(Sharma et al., 2021d)[64]	United States.	Use of new technology for social connectedness during the COVID-19 pandemic.	382 participants, balanced gender distribution, average age 43.9 years, predominantly White (71.2%).	Cross-sectional study using a 40-item questionnaire based on the Multi-Theory Model (MTM).	Initiation of New Technology Use for Social Connectedness: In this study, the initiation of new technology use among participants during the COVID-19 pandemic was significantly predicted by participatory dialogue ($p < 0.05$), behavioral confidence ($p < 0.001$), and changes in the physical environment ($p < 0.05$), accounting for 38.3% of the variance. Sustenance of New Technology Use for Social Connectedness: For the sustenance of new technology use, significant predictors were emotional transformation ($p < 0.001$), practice for change ($p < 0.001$), and changes in the social environment ($p < 0.001$),

					explaining 42.6% of the variance.
(Olatunde et al., 2022)[65]	The study was conducted among students at a large university in the United States.	The study focused on telehealth-based psychological help-seeking behaviors among college students.	The study targeted college students at a large U.S. university. A total of 356 students participated, with the average age being 24.31 years.	The study was a cross-sectional and survey-based research.	Initiation of Telehealth Among Past Users: For past telehealth users, participatory dialogue (advantages outweighing disadvantages) with a p-value of .009 and behavioral confidence with a p-value of <.001 were significant predictors for initiation. The variance explained in this behavior was 35.0%. Sustenance of Telehealth Among Past Users: In the sustenance model for past users, changes in the social environment were a significant predictor with a p-value of .016. The variance accounted for in this behavior was 23.0%. Initiation of Telehealth Among Non-Users: Among non-users, significant predictors for initiation were participatory dialogue (advantages outweighing disadvantages) with a p-value of <.001 and behavioral confidence with a p-value of .027. The variance in initiation behavior for this group was 16.9%. Sustenance of Telehealth Among Non-Users: For non-users, previous in-person treatment emerged as a significant predictor for

					sustenance with a p-value of .001. The variance explained in sustenance behavior was 14.4%.
(Wells et al., 2023)[66]	United States.	Intentions to use telehealth/telemedicine among veterinarians.	The study involved 243 veterinarians.	Cross-sectional study.	Initiation of Telehealth Use Among Veterinarians: In the final model for initiation, none of the Multi-Theory Model (MTM) constructs were found to be significant. The model explained 8.4% of the variance in the initiation of telehealth use among veterinarians. Sustenance of Telehealth Use Among Veterinarians: For the sustenance of telehealth use, emotional transformation was the only significant MTM construct with a p-value of <0.001. The model explained 25% of the variance in sustaining telehealth use among veterinarians.
(Panjwani et al., 2021)[67]	Mangaluru, Karnataka, India.	Toothbrushing behavior among dental and medical students.	224 undergraduate students pursuing medicine and dentistry.	Exploratory cross-sectional study using a modified MTM questionnaire.	The study assessed the MTM's adaptability in predicting toothbrushing behavior. It concluded that the Indian version of MTM is a reliable and valid instrument for measuring the initiation and sustenance of toothbrushing behavior among these students.
(Panjwani et al., 2022)[68]	Mangaluru, Karnataka, India.	The study focused on the initiation and sustenance of twice-daily teeth brushing behaviors.	The study targeted undergraduate students from	An analytical cross-sectional study.	Initiation of Twice-Daily Teeth Brushing Behavior: The study

			year one to year five pursuing Medicine and Dentistry at a university. A total of 229 students participated in the study.		conducted a hierarchical multiple regression analysis to understand the factors influencing the initiation of twice-daily teeth brushing among students. Behavioral confidence was identified as a significant predictor, contributing to 12.4% of the variance in this behavior with a p-value of <0.001. Subsequently, the addition of academic progression as a variable explained an additional 15.6% of the variance. Furthermore, incorporating sleeping habits into the model accounted for an extra 17.2% of the variation. Overall, these three variables combined explained a total of 45.2% of the variance in the initiation of twice-daily teeth brushing behavior, with each change in the model showing significant p-values of <0.001. The aspect of sustenance of this behavior was not explicitly included in their analysis.
(Sharma et al., 2022f)[69]	United States.	Flossing behavior among minority adolescents.	The study targeted African American/Black and Latinx/Hispanic adolescents aged 10–17 years, with a total of 520 participants.	Cross-sectional and analytical study.	Initiation of Flossing Behavior Among Participants Who Were Not Flossing: In the final model for these participants, behavioral confidence was a significant predictor with a p-value of <0.001.

					This model explained 33.5% of the variance in the initiation of flossing behavior. Sustenance of Flossing Behavior Among Participants Who Were Not Flossing: For the sustenance of flossing behavior among these participants, the final model identified emotional transformation ($p < 0.05$), practice for change ($p < 0.001$), and changes in the social environment ($p < 0.05$) as significant predictors. The model explained 37.8% of the variance in sustaining flossing behavior.
(Knowlden et al., 2017)[70]	A large Southern public university in the United States.	Initiation and sustenance of adequate sleep behavior in college students.	College students at the university, with a total of 151 participants. The study focused on those who were not currently receiving adequate sleep.	Cross-sectional study using a survey based on the Multi-Theory Model (MTM) of health behavior change.	Initiation of Adequate Sleep Behavior: The initiation model revealed that behavioral confidence was the only significant predictor, explaining 24.4% of the variance in the behavioral initiation of adequate sleep behavior. The significance of behavioral confidence was strong, with a p-value of <0.001. Sustenance of Adequate Sleep Behavior: In the sustenance model, several predictors were significant. Emotional transformation ($p = 0.001$), practice for change ($p < 0.001$), and changes in the social environment ($p = 0.023$) were all

					found to be significant predictors of behavioral sustenance for adequate sleep behavior. Collectively, these constructs accounted for 34.2% of the variance in sustaining adequate sleep behavior.
(Sharma et al., 2020b)[71]	Dental college in India.	Inadequate sleep patterns.	535 dental students, predominantly female (74.4%).	Cross-sectional survey.	Initiation of Adequate Sleep Behavior: In this study, the initiation of adequate sleep behavior among Indian dental students was significantly predicted by behavioral confidence. This factor was associated with a p-value of 0.019 and contributed to an adjusted variance of 3.9% in the model. Sustenance of Adequate Sleep Behavior: For the sustenance of adequate sleep behavior, emotional transformation emerged as a significant predictor, with a p-value of 0.047. It explained an adjusted variance of 9.0% in the model's predictive capacity.
(Sharma et al., 2017)[72]	A large public university in the Southeastern United States.	Initiating and sustaining the consumption of plain water instead of sugar-sweetened beverages among college students.	College students reported consuming at least one sugar-sweetened beverage in the past 24 hours. The study included 174 participants.	Cross-sectional study using a Multi-Theory Model (MTM)-based survey.	Initiation of Plain Water Consumption: The significant predictors for the initiation of plain water consumption were behavioral confidence and changes in the physical environment, each

					with a p-value of 0.001. These predictors collectively explained 61.8% of the variance in the initiation of this behavior change. Sustenance of Plain Water Consumption: For the sustenance of plain water consumption, the significant predictors were emotional transformation and practice for change, both with p-values of 0.001. These factors accounted for 58.3% of the variance in sustaining the behavior change.
(Sharma et al., 2020a)[73]	National Capital Region (NCR) of India.	Change in sugar-sweetened beverage (SSB) consumption behavior among university students.	University students in the NCR of India, 267 participants.	Cross-sectional study using the Multi-Theory Model (MTM) for health behavior change.	Initiation of SSB Consumption Behavior Change: In the study, the significant predictors for the initiation of sugar-sweetened beverage (SSB) consumption behavior change among Indian university students were participatory dialogue and behavioral confidence. Participatory dialogue had a p-value of 0.004, while behavioral confidence had a p-value of <0.001. These predictors together explained 16.7% of the variance in the initiation of SSB consumption behavior change. Sustenance of SSB Consumption Behavior Change: For the sustenance of SSB consumption

					behavior change, the significant predictors were emotional transformation and changes in social environment. Emotional transformation had a p-value of <0.001, and changes in social environment had a p-value of 0.002. These factors accounted for 30.2% of the variance in the sustenance of SSB consumption behavior change.
(Sharma et al., 2021e)[74]	United States.	Mammography screening among Asian American women.	374 Asian American women aged 45–54 years.	Cross-sectional study using a 44-item questionnaire.	Initiation of Mammography Screening: In the study on Asian American women, participatory dialogue, behavioral confidence, and changes in the physical environment were significant predictors for initiating mammography screening. These factors explained 49.9% of the variance in decision-making, with p-values of <0.05 for participatory dialogue and <0.001 for both behavioral confidence and changes in the physical environment. Sustenance of Mammography Screening: For sustaining mammography screening, emotional transformation and practice for change were identified as significant predictors,

					accounting for 53.9% of the variance. Both factors had p-values of <0.001.
(Sharma et al., 2022c)[75]	United States.	Mammography screening behavior among Hispanic American women.	Hispanic women aged 45-54 years.	Cross-sectional study using a 50-item web-based survey.	Initiation of Mammography Screening: In the final model for initiation of mammography screening, participatory dialogue and changes in the physical environment were significant predictors with p-values < 0.05, while behavioral confidence had a p-value < 0.001. This model accounted for 33.4% of the variance. Sustenance of Mammography Screening: For the sustenance model, only practice for change and changes in the social environment were significant predictors, both with p-values < 0.001, explaining 52.7% of the variance.
(Sharma et al., 2022a)[76]	United States.	Cervical cancer screening (Pap test) among minority women.	364 minority women.	Cross-sectional study using a survey.	Initiation of Cervical Cancer Screening: In the study, participatory dialogue, behavioral confidence, and changes in the physical environment were significant predictors for initiating cervical cancer screening. Participatory dialogue and changes in the physical environment had a p-value of <0.05, and behavioral confidence had a p-value of <0.001.

					These predictors accounted for 49.5% of the variance in initiating Pap test behavior. Sustenance of Cervical Cancer Screening: Emotional transformation, practice for change, and changes in the social environment were significant predictors for sustaining Pap test behavior every three years. Emotional transformation and practice for change had p-values of <0.001, changes in the social environment had a p-value of <0.05. These factors explained 73.6% of the variance.
(Sharma et al., 2023)[77]	The study was conducted across the United States.	The research focused on the behavior of seeking stool-based tests for colorectal cancer (CRC) screening.	The study included 640 U.S. adults aged 45–75 years who understand English. The sample was diverse, representing the current U.S. population demographics.	This was a cross-sectional study utilizing an online 57-item questionnaire with established psychometric validity to collect responses.	Among Individuals Who Have Never Had Any Form of Colorectal Cancer Screening: In this group, the model accounted for 48.1% variance in the intent to seek stool-based CRC screening tests. The significant constructs influencing this behavior were participatory dialogue, behavioral confidence, and changes in the social environment, each with a p-value of <0.05. Among Individuals Who Have Had Any Form of Colorectal Cancer Screening in the Past: For this group, the model accounted for 60.9% variance in the intent to seek

					stool-based CRC screening tests. The significant constructs influencing this behavior were participatory dialogue, behavioral confidence, and changes in the social environment, each with a p-value of <0.001.
(Sharma et al., 2018b)[78]	A large public university in the Southeastern United States.	Initiating and sustaining responsible drinking or abstinence among college students who binge drink.	College students who binge drank within the past 30 days. A total of 289 students participated in the study.	Cross-sectional study utilizing a Multi-Theory Model (MTM) approach.	Initiation of Responsible Drinking or Abstinence: The study identified race/ethnicity (p = 0.004), behavioral confidence (p = 0.029), and changes in the physical environment (p = 0.001) as significant predictors in the initiation model, accounting for 20% of the variance in the intention to start responsible drinking or abstinence. Sustenance of Responsible Drinking or Abstinence: For sustaining this behavior, the significant predictors in the final model were race/ethnicity (p < 0.001) and practice for change (p < 0.001), also explaining 20% of the variance in the continuation of responsible drinking behaviors.
(Claros et al., 2020)[79]	Mental health clinic-based setting.	Substance use behavior change.	93 participants who completed treatment at a substance use treatment facility, majority Caucasian (72.7%) and male (60%).	Cross-sectional design using a newly developed self-administered questionnaire.	Initiation of Substance Use Cessation: The study identified participatory dialogue (advantages minus disadvantages) and behavioral confidence as

					significant predictors for initiating substance use cessation. These predictors accounted for a total variance of 34.2%. Participatory dialogue had a p-value of 0.001, and behavioral confidence had a p-value of less than 0.001. Sustenance of Substance Use Cessation: For the sustenance of substance use cessation, the significant predictors were practice for change and changes in the social environment, accounting for 33.0% of the total variance. The p-values for practice for change and changes in the social environment were 0.004 and less than 0.003, respectively.
(Sharma et al., 2021a)[80]	Large public university in the Southern U.S.	Handwashing behavior among college students during the COVID-19 pandemic.	713 college students, 70.3% female, average age 24.61 years, predominantly white (74.3%).	Cross-sectional survey using the Multi-Theory Model (MTM) of health behavior change.	Initiation of Handwashing Behavior: The significant predictors were behavioral confidence ($p<0.01$) and participatory dialogue ($p<0.05$), explaining 27.2% of the variance in the behavior among those not following handwashing recommendations. Sustenance of Handwashing Behavior: Emotional transformation ($p<0.01$), practice for change ($p<0.01$), and changes in the social environment ($p<0.05$) were

					significant predictors, accounting for 45.1% of the variance in the likelihood of maintaining handwashing behavior.
(Sharma et al., 2021b)[81]	Southeastern United States, private sleep center.	Adherence to Positive Airway Pressure (PAP) therapy in newly diagnosed sleep apnea patients.	138 newly diagnosed sleep apnea patients prescribed PAP therapy.	Cross-sectional study using a 41-item Multi-Theory Model (MTM) instrument.	Initiation of PAP Therapy Adherence: The study examined the initiation of positive airway pressure (PAP) therapy adherence among newly diagnosed sleep apnea patients. The significant predictors for initiation were participatory dialogue, behavioral confidence, and changes in the physical environment, contributing to 53.5% of the variance in initiating PAP therapy. Participatory dialogue had a p-value of 0.014, behavioral confidence had a p-value of <0.001, and changes in the physical environment had a p-value of 0.001. Sustenance of PAP Therapy Adherence: For sustaining PAP therapy adherence, emotional transformation, changes in the social environment, and practice for change were the significant predictors. These factors accounted for 60.6% of the variance in the sustenance of PAP therapy adherence.

					Emotional transformation had a p-value of <0.001, changes in the social environment had a p-value of 0.016, and practice for change had a p-value of 0.047.
(Davis et al., 2021)[82]	Large public university in the Southeastern U.S.	Mask-wearing behaviors among college students during COVID-19.	595 college students, predominantly female (73.4%) and White (71.2%).	Cross-sectional electronic survey.	Initiation of Mask-Wearing Behavior: In the study by Davis et al., the significant predictors for initiating mask-wearing behavior among college students were participatory dialogue (advantages-disadvantages) with a p-value of 0.010, behavioral confidence with a p-value of <0.001, and changes in the physical environment with a p-value of <0.001. These factors accounted for 34.6% of the variance in initiation. Sustenance of Mask-Wearing Behavior: For sustaining mask-wearing behavior, significant predictors were emotional transformation with a p-value of <0.001, practice for change with a p-value of 0.017, and changes in the social environment with a p-value of 0.029, explaining 33.0% of the variance in sustenance.
(Sharma et al., 2021g)[83]	Florida, USA.	Sunscreen usage among Florida residents.	General population, 1284 participants, various demographics.	Cross-sectional study using a web-based survey.	Initiation of Sunscreen Use: The study found that participatory dialogue, behavioral confidence, and

					changes in the physical environment were significant predictors for initiating sunscreen use among Florida residents. These predictors accounted for 73.6% of the variance in the initiation of sunscreen use, with p-values of <0.05 for participatory dialogue and <0.001 for both behavioral confidence and changes in the physical environment. Sustenance of Sunscreen Use: For sustaining sunscreen use, emotional transformation, practice for change, and changes in the social environment were identified as significant predictors, explaining 59% of the variance. Each of these factors had a p-value of <0.001.
(Sharma et al., 2021h)[84]	United States.	Meditation intention among U.S. adults.	330 U.S. adults, with a significant proportion of older adults (mean age 65.02 years), predominantly White (92.8%), and unemployed (71.3%).	Cross-sectional study using a web-based survey.	Initiation of Meditation Behavior: The study identified participatory dialogue and behavioral confidence as significant predictors for initiating meditation behavior, accounting for 39.5% of the variance. Participatory dialogue had a p-value of 0.002, and behavioral confidence had a p-value of less than

					0.001. Sustenance of Meditation Behavior: For sustaining meditation behavior, emotional transformation and changes in the social environment were significant predictors, explaining 60.4% of the variance. Emotional transformation had a p-value of less than 0.001, and changes in the social environment had a p-value of less than 0.001.
(Hayes et al., 2022)[85]	Jackson State University, Jackson, Mississippi, United States.	Yoga practice among college students.	70 college students, mean age 28.62 years, predominantly female (84%) and black (87%).	Cross-sectional study using a 36-item questionnaire.	Initiation of Yoga Practice: In Hayes et al.'s study, the initiation of yoga practice among college students was significantly predicted by changes in the physical environment (p = .002) and behavioral confidence (p = .008), explaining 40% of the variance in initiating yoga practice. Sustenance of Yoga Practice: For maintaining yoga practice, the study identified practice for change as a significant predictor, accounting for 46% of the variance (p = .0001).
(Sharma et al., 2022e)[86]	Northern India.	Screen time (ST) behavior among preschoolers.	The study involved a convenience quota sample of 72 North Indian parents of 2 to 5-year-old preschoolers.	Cross-sectional survey.	Initiation of Reducing Screen Time Behavior: The final model explained 33.4% of the variance in the initiation of reducing screen time behavior. The significant predictors were

					behavioral confidence and changes in the physical environment, both with a p-value of <0.001. Sustenance of Reducing Screen Time Behavior: The final model for the sustenance of reducing screen time behavior explained 39.7% of the variance. The significant predictors were emotional transformation ($p < 0.001$), practice for change ($p < 0.001$), and changes in the social environment ($p = 0.001$).
(Wilkerson et al., 2023)[87]	The study was conducted at two large public universities in the south-central and southeastern United States.	The research examined the behavior of indoor tanning cessation among college students.	The study included 254 college students who were current indoor tanners. The average age was 22.08 years, with a majority being female (90.6%) and White (87.4%).	This was a cross-sectional study using a validated survey to assess the Multi-Theory Model (MTM) constructs.	Initiation of Indoor Tanning Cessation: The model accounted for 60.3% variance in the initiation of indoor tanning cessation. Participatory dialogue: advantages, behavioral confidence, and changes in the physical environment were significantly associated with the initiation of indoor tanning cessation, each having a p-value of <0.001. Sustenance of Indoor Tanning Cessation: For the sustenance of indoor tanning cessation, the model accounted for 44.7% variance. Emotional transformation was the only construct that exhibited a significant relationship, with a p-value of <0.001.

(Kapukotuwa et al., 2023)[88]	The study was conducted at a large university in the southwestern region of the United States.	The research examined gambling behavior among university students.	The study included university students who were current or past participants in gambling activities.	This was a cross-sectional study employing a survey to assess the Multi-Theory Model (MTM) constructs.	Initiation of Quitting Gambling Behavior: The model accounted for 27.7% variance in the likelihood of initiating the behavior change. Participatory dialogue, behavioral confidence, and changes in the physical environment were the significant constructs for initiation, with p-values of <0.0001, <0.0001, and 0.0137, respectively. Sustenance of Quitting Gambling Behavior: The model accounted for 22.6% variance in the likelihood of sustaining quitting gambling behavior. Emotional transformation and practice for change were the significant constructs for sustenance, each with p-values of 0.0003 and 0.0368, respectively.
